# Engagement With a Behavior Change App for Alcohol Reduction: Data Visualization for Longitudinal Observational Study

**DOI:** 10.2196/23369

**Published:** 2020-12-11

**Authors:** Lauren Bell, Claire Garnett, Tianchen Qian, Olga Perski, Elizabeth Williamson, Henry WW Potts

**Affiliations:** 1 Department of Medical Statistics London School of Hygiene and Tropical Medicine London United Kingdom; 2 Research Department of Behavioural Science and Health University College London London United Kingdom; 3 Department of Statistics University of California Irvine Irvine, CA United States; 4 Health Data Research UK London United Kingdom; 5 Institute of Health Informatics University College London London United Kingdom

**Keywords:** mobile health, behavior change, apps, digital health, data visualizations, engagement, micro-randomized trial, push notifications, just-in-time adaptive interventions

## Abstract

**Background:**

Behavior change apps can develop iteratively, where the app evolves into a complex, dynamic, or personalized intervention through cycles of research, development, and implementation. Understanding how existing users engage with an app (eg, frequency, amount, depth, and duration of use) can help guide further incremental improvements. We aim to explore how simple visualizations can provide a good understanding of temporal patterns of engagement, as usage data are often longitudinal and rich.

**Objective:**

This study aims to visualize behavioral engagement with *Drink Less*, a behavior change app to help reduce hazardous and harmful alcohol consumption in the general adult population of the United Kingdom.

**Methods:**

We explored behavioral engagement among 19,233 existing users of *Drink Less*. Users were included in the sample if they were from the United Kingdom; were 18 years or older; were interested in reducing their alcohol consumption; had a baseline Alcohol Use Disorders Identification Test score of 8 or above, indicative of excessive drinking; and had downloaded the app between May 17, 2017, and January 22, 2019 (615 days). Measures of when sessions begin, length of sessions, time to disengagement, and patterns of use were visualized with heat maps, timeline plots, k-modes clustering analyses, and Kaplan-Meier plots.

**Results:**

The daily 11 AM notification is strongly associated with a change in engagement in the following hour; reduction in behavioral engagement over time, with 50.00% (9617/19,233) of users disengaging (defined as no use for 7 or more consecutive days) 22 days after download; identification of 3 distinct trajectories of use, namely engagers (4651/19,233, 24.18% of users), slow disengagers (3679/19,233, 19.13% of users), and fast disengagers (10,903/19,233, 56.68% of users); and limited depth of engagement with 85.076% (7,095,348/8,340,005) of screen views occurring within the *Self-monitoring and Feedback* module. In addition, a peak of both frequency and amount of time spent per session was observed in the evenings.

**Conclusions:**

Visualizations play an important role in understanding engagement with behavior change apps. Here, we discuss how simple visualizations helped identify important patterns of engagement with *Drink Less*. Our visualizations of behavioral engagement suggest that the daily notification substantially impacts engagement. Furthermore, the visualizations suggest that a fixed notification policy can be effective for maintaining engagement for some users but ineffective for others. We conclude that optimizing the notification policy to target both effectiveness and engagement is a worthwhile investment. Our future goal is to both understand the causal effect of the notification on engagement and further optimize the notification policy within *Drink Less* by tailoring to contextual circumstances of individuals over time. Such tailoring will be informed from the findings of our micro-randomized trial (MRT), and these visualizations were useful in both gaining a better understanding of engagement and designing the MRT.

## Introduction

### Background

Maintaining alcohol consumption within recommended guidance is widely known to reduce one’s risk of illness or injuries. Such guidance includes the recommendations of the Chief Medical Officer of the United Kingdom to limit alcohol consumption to 14 units a week and to have frequent alcohol-free days [1]. However, anyone in the general adult population who wants to reduce their hazardous or harmful alcohol consumption may face certain challenges to follow such guidance [2]. Challenges include the ease of access to alcohol and alcohol being an addictive substance. This can lead to individuals developing chronic or cyclical patterns of excessive drinking, with the personal behaviors of drinking influenced by internal or external factors [3-5]. Internal factors refer to feeling states or events in an individual’s recent drinking history, such as previous drinking episodes, moods, motives, or cravings that may modify future patterns of drinking [6]. External factors are influential events that occur independently of an individual’s drinking history; for example, how the risk of hazardous drinking of the general population increases during holiday periods or weekends [7,8].

Behavior change apps, sensors, and wearables offer a way of reducing hazardous alcohol consumption through real-time data capture and interventions [9-12]. Benefits of behavior change apps, that can be synchronized with sensors and wearables, include capturing an individual’s dynamic history of alcohol consumption and state of mind while providing *around the clock* access to support, particularly in moments when an individual’s vulnerability to hazardous drinking may increase [13].

However, a key challenge for the majority of behavior change apps is that levels of engagement remain low [14-16]. Engagement, often a mediator of effectiveness [14], is considered a multifaceted construct composed of behavioral and experiential aspects [17]. Usage data from a behavior change app provides an understanding of behavioral engagement (hereafter referred to as engagement) with the app [18]. Multiple indicators of engagement are thought to convey important information about how users interact with a given intervention, including the frequency (eg, number of log-ins), depth (eg, proportion of available modules accessed), amount (eg, time spent per log-in), and duration (eg, total number of days) of use [19].

*Drink Less* is a behavior change app that aims to help its users reduce hazardous and harmful alcohol consumption. The app was developed following the multiphase optimization strategy framework (comprising a preparation phase, an optimization phase, and an evaluation phase) [20-23] and the UK Medical Research Council’s guidance on developing complex interventions [24-26]. The app includes 6 different theory and evidence-informed modules: normative feedback, goal setting, cognitive bias training, self-monitoring and feedback, action planning, and identity change. These modules are described in detail by Garnett et al [27]. The app sends a local daily push notification at 11 AM that asks users to “Please complete your drink diaries,” to encourage self-monitoring of drinking behavior. The default 11 AM timepoint was set so as not to disturb late risers and to allow participants time to complete their morning routine; however, the notification timing could be changed by the user.

Owing to the agile nature of app development, optimization of engagement can be done through cycles of research and implementation [28]. Identifying important patterns of engagement for such optimization purposes presents various analytical challenges that visualizations can address. Visualizations have previously been helpful for analyzing a wide variety of rich data streams within public health research [29-33]. Simple visualizations, especially when complemented with clear textual descriptions, are generally recommended for identifying and comparing trends [32]. In previous digital health research, visualizations have delivered *at a glance* insight from mass volume and time-varying data, including more sophisticated displays of spatiotemporal, contextual, and event-centric outcomes [34-38]. Importantly, visualizations can provide insights into optimization that include (1) patterns of use that may boost or hinder behavior change, (2) a better understanding of temporal engagement with various components of the intervention, and (3) pathways toward personalization of the intervention.

### Objectives

The aim of this paper is to explore the usefulness of simple visualizations in uncovering important temporal patterns of engagement and facilitating decision making for further intervention development. This study presents 2 key contributions to improving engagement with *Drink Less*. The first contribution, provided in the Results section, is to showcase a number of visualizations that helped us understand temporal patterns of engagement with *Drink Less*. The second contribution, provided in the Discussion section, explains how insights obtained from these visualizations informed the next stages of intervention optimization.

## Methods

### Data Transformation

Each visualization involved transformation of the data. Original usage data involved merging, by an anonymous user ID, a data set of baseline characteristics (age, sex, employment type, and Alcohol Use Disorders Identification Test [AUDIT] score) to a data set of time stamps of start time of use, screen views, and length (in microseconds) of use. Along with use, the actions of entering an alcohol-free day or recording units of alcohol consumed were measured.

### Data

Data set 1 included 19,233 users who downloaded *Drink Less* between May 17, 2017, and January 22, 2019 (615 days). The inclusion criteria for users included having a baseline AUDIT score of 8 or above, which is indicative of excessive drinking [39]; being from the United Kingdom; being aged 18 years or above; being interested in reducing their alcohol consumption; using app versions 1.0.11 to 1.0.16; and having consented to the Privacy Notice (Multimedia Appendix 1). Screen views data are recorded automatically and downloaded via Panda scripts from *Nodechef* (a web-based platform for hosting mobile apps) using a secure https protocol. Sessions were derived from screen views using the Pandas script.

Users who downloaded the app on August 21, 2018 (n=5830), were excluded as an article on BBC News was published on this date, which endorsed the app (Garnett et al, unpublished data, 2020); thus, these users were likely to have different characteristics and engagement behavior.

Data set 2 included time stamps of 829,001 sessions and 8,169,005 screen views of the 19,233 users in data set 1. This includes 122,332 entries of alcohol-free days and 123,704 entries of alcohol drinks consumed. All use was recorded from May 17, 2017, to April 16, 2019 (699 days). As such, users had a minimum of 84 days of use measured.

To explore various engagement aspects, we developed sets of data from data sets 1 and 2 with varying engagement measures.

#### Set A

All use was measured from May 17, 2017, to January 22, 2019 (615 days), including date of download and time stamps of all use. This period was chosen as it reflects a time in which the content of the app was relatively stable.

#### Set B

Set B included all users whose use was measured in Set A, with data only over the first 30 days from download, with the measure “Did use occur on this day?” (binary, yes or no) for each user.

### Measures

#### Log-in Sessions and Frequency of Log-Ins

A session was defined as a continuous series of screen views, with a new session defined as a new screen view after 30 min of inactivity [40]. Clearing or *swiping away* the daily notification did not register as use and was not considered as either a session or a module view. All time stamps were appropriately adjusted from Coordinated Universal Time to British Summer Time. The amount of use per log-in session was operationalized as time spent (in seconds) per session. Daily use was captured by the measure “Did use occur on this day?” (binary, yes or no) for each user for 30 days (Set B).

#### Drinking Diary Entry

In the self-monitoring and feedback module, users enter an alcohol-free day and the date of its occurrence, the number of alcoholic units consumed, and the date of consumption. The time stamps in which records were made was measured.

#### Disengagement

We defined disengagement as the first day of 7 or more consecutive days of no use after download [41]. The days between download and disengagement were derived for each user. Users who did not disengage after downloading the app were censored.

### Data Visualization Methods and Analytical Techniques

We used heat maps, timeline plots, k-modes clustering, generalized estimation equations, and Kaplan-Meier plots to explore and visualize patterns of engagement with *Drink Less*. Analyses were carried out in R [42] and Stata [43]. We used the following R library packages to create the visualizations: ggplot2 for heat maps and timeline plots [44], rayshader to create the 3D animations [45], viridis for color palettes sensitive to readers with color blindness [46], Klar to perform the k-modes clustering [47], survminer for the Kaplan-Meier survival curves and number at risk table [48], gganimate to create animated plots of use over time [49], and patchwork to place graphs side by side [50]. The data visualization methods, data set and engagement measures are shown in Textbox 1.

K-modes clustering is an extension of the k-means algorithm for partitioning categorical data, which uses a general dissimilarity measure [51,52]. Within each cluster, we visualized the probability of opening the app during the day over time with 95% CI. The appropriate number of clusters was explored through the *elbow* method and *silhouette* method [53]. The *elbow* method explains the variance of the data in relation to the number of clusters and shows by how much the addition of another cluster would reduce the dissimilarity measure. The *silhouette* method shows how well each user fits into their respective cluster through 2 distance measures: separation (ie, the average distance to the closest other cluster) and compactness (ie, the average within-cluster distance) [54,55]. Kaplan-Meier plots show the estimated cumulative proportion of users engaged and the time scale is days after download [56,57].

**Data visualization methods, data, and engagement measures.**

Set A:

- Heat maps: Total count of sessions and total amount of time spent on *Drink Less*, by hour and day of the week
- Timeline plots: Frequency and median amount of time per session
- Kaplan-Meier plots: Time to disengagement (defined as days after download followed by 7 or more consecutive days of nonuse)

Set B:

- K-modes clustering: Was the app used or not each day, over 30 days after download

To explore the association between the delivery of the notification and subsequent near-term engagement of opening the app (ie, engagement in the hour after the notification is delivered), we compared opening the app (yes or no) between the *exposed* time period (11 AM to noon) and an *unexposed* time period (10 AM to 11 AM). We estimated the association between exposure to the notification and opening of the app, which was quantified using a risk ratio. We fitted a marginal model for the outcome of opening the app by using a generalized estimating equation [58] with robust standard errors and an independent working correlation matrix. We fitted an unadjusted model and a model adjusted for the baseline covariates of the continuous variables age, days after download and baseline AUDIT score, which were all included as linear terms, and the categorical variables employment type and gender. Further models explored effect moderation by adding an interaction between exposure to the notification and (1) days after download and (2) cluster (as identified by the k-modes analysis). In the final model, we additionally allowed the association between cluster and exposure to the notification to vary linearly by day after download. Estimated risk ratios with 95% CIs and Wald test *P* values are presented. For models with interaction terms, we present risk ratios for exposure to the notification estimated at days 1, 7, and 30 after download, estimated separately for each cluster.

## Results

### Overview

The user characteristics are reported in Table 1. Approximately half (49.5%) of the sample were male. The mean age of users was 44 (SD 11.2) years, and the majority worked in nonmanual employment (71.7%). Just under half (46.6%) had a baseline AUDIT score indicating hazardous alcohol consumption (8 to 15, inclusive).

**Table 1:** User characteristics (N=19,233).

User characteristics		Participants
**Sex, n (%)**		
	Male	9540 (49.60)
Age (years), mean (SD)		44 (11.2)
**Employment type, n (%)**		
	Nonmanual employment	13,792 (71.71)
**AUDIT^a^ risk zone, n (%)**		
	Hazardous (8-15)	8958 (46.58)
	Harmful (16-19)	3949 (20.53)
	At risk of alcohol dependence (20-40)	6326 (32.89)

^a^AUDIT: Alcohol Use Disorders Identification Test.

Summative tables of use (screen views and time on app) by module are provided in Multimedia Appendix 2. It was observed that 85% of screen views occurred in the module *Self-Monitoring and Feedback*. The number of users who reported at least one alcohol-free day or at least one alcohol drink record was 61.86% (11,898/19,233) and 49.11% (9445/19,233), respectively. Over the first 30 days of use after download (derived for Set B data), the median number of sessions per user was 9, with an IQR of 2 to 28 sessions, and the median time spent per user was 24 min, with an IQR of 9 to 55 min.

### Visualizations

#### Patterns of Frequency of Use, Length of Use, Entries of Alcohol-Free Days, and Alcohol Units Consumed

**Figure 1:**
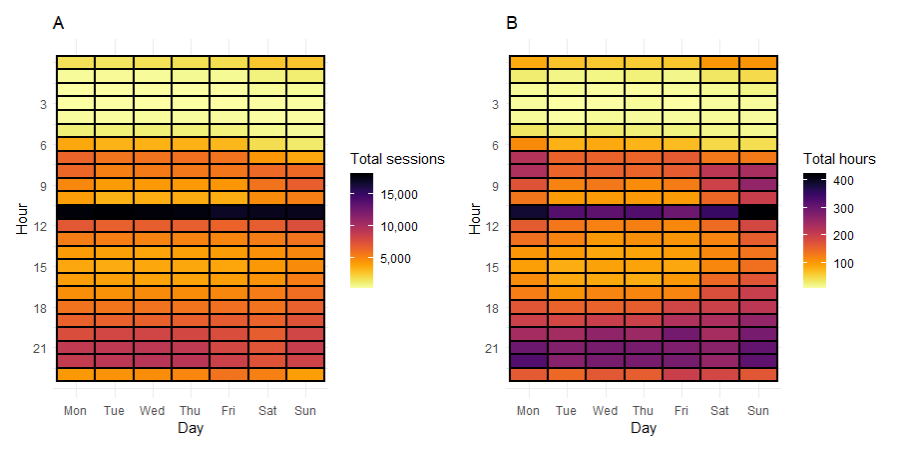
Heat maps of total frequency of use (sessions) and total time on app (hours).

In Figure 1, both heat maps show days of the week along the x-axis and hour of the day along the y-axis. Plot A in Figure 1 shows the frequency of opening the app by hour of the day and day of the week. This shows that there is a strong association between delivery of the notification and opening of the app in the following hour, and this is consistent throughout the week. Plot B in Figure 1 shows the amount of use by hour of the day and day of the week. This shows that the notification is also associated with the distribution of the total time spent on the app. In plot B, hotspots are observed across the evenings and on Saturday, Sunday, and Monday mornings, which are not evident in plot A. A heat map of when *Drink Less* was downloaded (Multimedia Appendix 3) shows hotspots of downloads on Sunday and Monday evenings. Rotating 3D heat map films of Figure 1, which show the variations more clearly, are provided in Multimedia Appendices 4 and 5.

**Figure 2:**
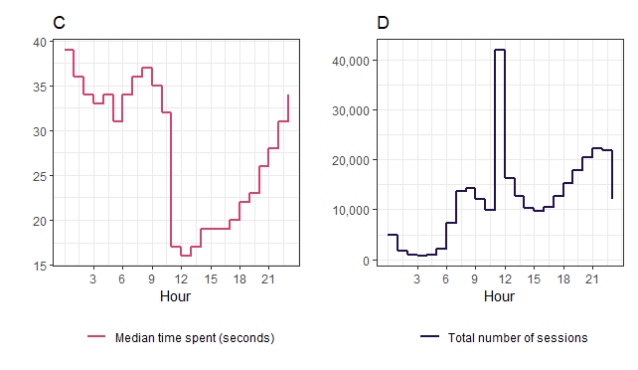
Median time spent on the *Drink Less* app per session and frequency distribution of sessions.

In Figure 2, plot C shows the median time spent on the app along the y-axis and plot D shows the total number of sessions starting in the hour along the y-axis. Timeline plots show the hour of the day on the x-axis. Plot D shows that the frequency of sessions sharply peaks in the hour after the notification is sent at 11 AM. A second natural peak of frequency occurred in the evenings and a third smaller peak in the mornings. Plot C shows that the median length of time drastically dropped from 11 AM onward, with a slow and steady recovery as the day progressed. An animation of plot D over time is provided in Multimedia Appendix 6, showing that the shape of the distribution over 30 days remains consistent.

**Figure 3:**
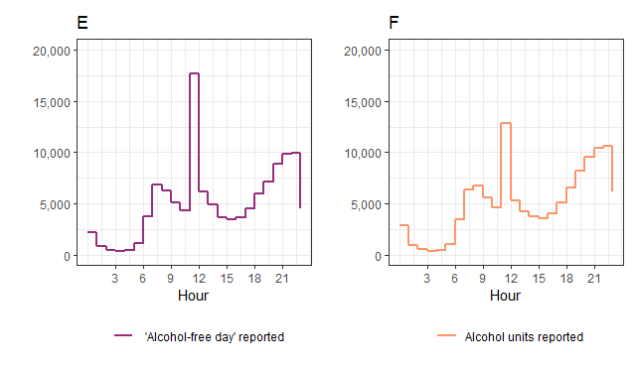
Frequency distributions of when alcohol-free days and alcohol units are recorded during the day.

In Figure 3, plot E shows the frequency distribution of entering an alcohol-free day and plot F shows the frequency of entering a drink record. Timeline plots show the hour of the day on the x-axis. There are more alcohol-free days entered between 11 AM to 12 PM than drink records made, which suggests that the notification is more strongly associated with entering *alcohol-free days* than entering *alcohol units consumed*. Both outcomes see similar prominent, natural peaks in the evenings, with an additional smaller peak in the mornings.

#### Visualization of Engagement Clusters

A total of 3 clusters emerged from the k-modes clustering. This was based on the measure *did the user open the app?* (binary, yes or no) for the first 30 days after download.

**Figure 4:**
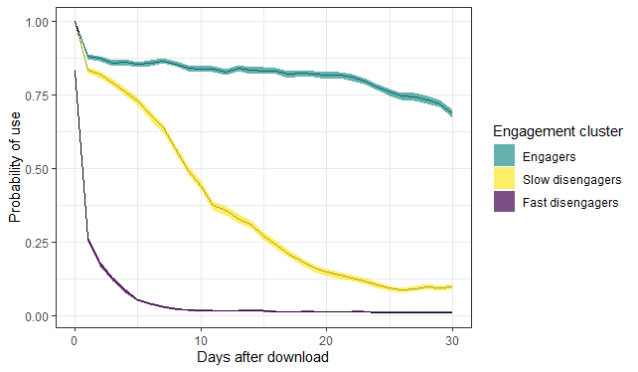
Probability of use on day after download by cluster group with 95% CIs.

Figure 4 plots the probability of use of the app, stratified by cluster, over time (number of days after download). The 3 ribbons represent the probability of use of the app for each engagement cluster, with 95% CI. On the basis of the observed pattern of engagement, we named the 3 clusters as fast disengagers (10,903/19,233, 56.68%), slow disengagers (3679/19,233, 19.12%), and engagers (4651/19,233, 24.18%). The optimal number of clusters was determined by the elbow method and silhouette method (Multimedia Appendix 7). The silhouette method suggested that the optimal number of clusters was 2, whereas the elbow method suggested 3 clusters. Comparing the results under 2 and 3 clusters showed that the slow disengagers and engagers groups identified under 3 clusters were essentially a subdivision of 1 cluster in the 2-cluster model. We chose to retain 3 clusters based on observed differences in the trajectory of engagement over time between the 2 groups—the engagers and slow disengagers.

The probability of using the app 30 days after download for engagers was 0.69 (95% CI 0.67-0.70), slow disengagers was 0.10 (95% CI 0.10-0.11), and fast disengagers was 0.01 (95% CI 0.01-0.02).

Table 2 shows the distribution of user characteristics across the engagement clusters. The median number of sessions was lowest for the fast disengagers and much higher for the engagers. Engagers are, on average, more likely to be older, male, working in nonmanual employment, and more likely to report a lower AUDIT risk zone, compared with users within the fast disengagers and slow disengagers clusters.

**Table 2:** User characteristics by cluster group.

User characteristics		Fast disengagers (n=10,903)	Slow disengagers (n=3679)	Engagers (n=4651)
Male, n (%)		4991 (45.78)	1920 (52.49)	2629 (56.53)
Age (years), mean (SD)		43.7 (11.57)	43.2 (10.91)	45.4 (10.57)
Employment type (nonmanual), n (%)		7567 (69.40)	2659 (72.28)	3566 (76.67)
**AUDIT^a^ risk zone, n (%)**				
	Hazardous (8-15)	5016 (46.01)	1577 (42.86)	2365 (50.85)
	Harmful (16-19)	2155 (19.77)	798 (21.69)	996 (21.41)
	At risk of alcohol dependence (20+)	3732 (34.23)	1304 (35.44)	1290 (27.74)
Number of sessions per user, median (25th-75th percentile)		3 (1-6)	18 (12-28)	88 (51-175)

^a^AUDIT: Alcohol Use Disorders Identification Test.

Table 3 provides the estimated associations between exposure to the notification and app use, based on Set B data. Over the first 30 days after day of download, the probability of using the app in the hour after the delivery of the notification was approximately 4 times higher than the probability of using the app in the hour before. All models of the estimated associations between exposure to the notification and app use are adjusted for the continuous variables of age, days after download, baseline AUDIT score, and the categorical variables of employment type and sex. The cluster-specific effects included an effect moderation of the exposure to the notification by cluster group, and the days after download effects included an effect moderation of the exposure by days after download. The adjusted estimated risk ratio was 4.21 (95% CI 4.07-4.36), and the estimated risk ratio was higher among engagers (Wald test *P* value: fast disengagers vs engagers *P*=.001 slow disengagers vs engagers *P*<.001, slow disengagers vs fast disengagers *P*=.44).

Table 4 shows the estimated association between exposure to the notification and opening of the app in the 3 clusters at 3 different time points (days 1, 7, and 30).

**Table 3:** Estimated associations between exposure to the notification and app use.

Model		Exposure to notification, estimated relative risk ratio (95% CI)
Unadjusted model		4.22 (4.13-4.31)
Adjusted model^a^		4.21 (4.07-4.36)
**Days after download^b^**		
	Day 1	3.93 (3.77-4.10)
	Day 7	4.07 (3.93-4.22)
	Day 30	4.67 (4.38-4.98)
**Cluster^c^**		
	Fast disengagers	3.97 (3.70-4.25)
	Slow disengagers	3.82 (3.60-4.03)
	Engagers	4.38 (4.18-4.59)

^a^Adjusted for days after download, employment type, sex, age, and baseline Alcohol Use Disorders Identification Test (AUDIT) score.

^b^Estimated from the model including the interaction effect of exposure to the notification by days after download, adjusted for employment type, sex, age, and baseline AUDIT score.

^c^Estimated from the model including the interaction effect of exposure to the notification by cluster, adjusted for days after download, employment type, sex, age, and baseline AUDIT score.

**Table 4:** Estimated risk ratio with 95% CI for the associations between exposure to the notification and app use within each cluster, at 3 time points (days 1, 7, and 30).

Clusters	Risk ratio at day 1 (95% CI^a^)	Risk ratio at day 7 (95% CI^a^)	Risk ratio at day 30 (95% CI^a^)
Fast disengagers	3.66 (3.33-4.02)	3.83 (3.57-4.11)	4.58 (3.86-5.43)
Slow disengagers	4.18 (3.85-4.54)	3.87 (3.64-4.12)	2.89 (2.43-3.43)
Engagers	4.05 (3.82-4.30)	4.22 (4.01-4.43)	4.90 (4.56-5.26)

^a^Interaction effect of exposure to the notification and days after download, an interaction effect of exposure to the notification and cluster, and a three-way interaction effect of exposure to the notification, cluster, and days after download, adjusted for employment type, sex, age, and baseline Alcohol Use Disorders Identification Test score.

#### Visualization of Time to Disengagement

Kaplan-Meier plots, both overall and stratified by clusters, were plotted to show days to disengagement, defined as 7 or more consecutive days of no use, for the first 365 days after downloading *Drink Less.*

**Figure 5:**
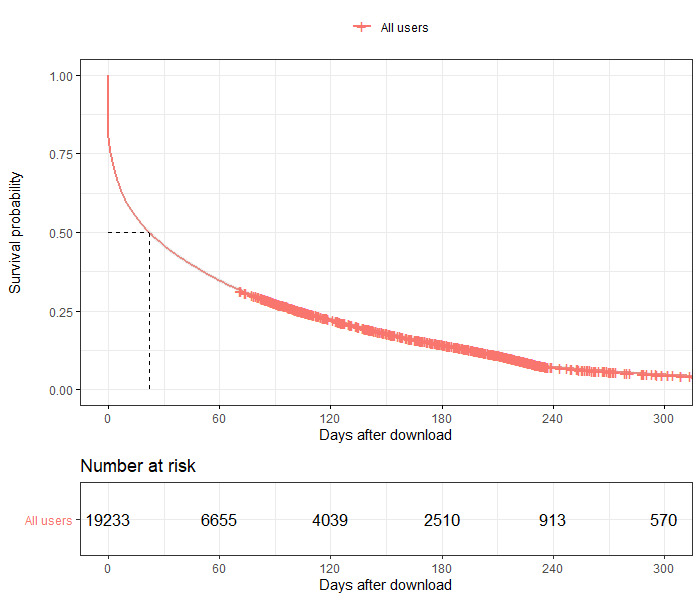
Time to disengagement (defined as the first day of 7 or more consecutive days of no use) for all users.

**Figure 6:**
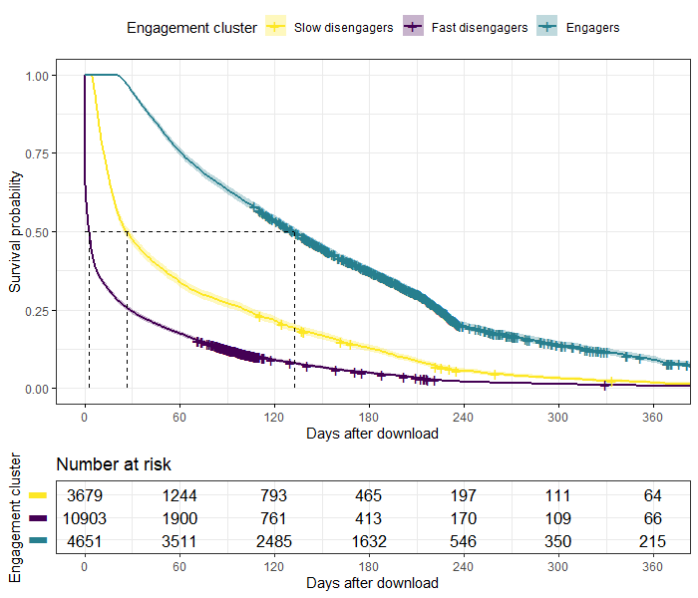
Time to disengagement (defined as the first day of 7 or more consecutive days of no use) by the engagement cluster.

In Figures 5 and 6, the x-axis depicts the number of days after download, ranging from 0 to 365, and the y-axis depicts the survival probability, which is the proportion of users who have not disengaged. The dashed lines at the 0.5 survival probability mark shows the time (days) up to when 50%of each cluster has disengaged. Each hash in the plot represents a right-censored user. The number at risk represents the users in the clusters who remain engaged over the year. In Figure 5, we see that 50.00% (9617/19,233) of users have disengaged at 22 days from download, and Figure 6 shows the divergence of longer-term engagement between clusters. The median number of days to disengagement for engagers was 132 days (95% CI 128-137), slow disengagers was 26 days (95% CI 24-29), and fast disengagers was 3 days (95% CI 2-3).

## Discussion

### Principal Findings

Visualizations provided important insights into how users engage with the behavior change app *Drink Less.* They revealed a strong association between delivery of the daily push notification (sent at 11 AM) and use in the next hour, suggesting that the push notification strongly influences how users engage with *Drink Less* in the immediate hour after the notification is sent. Push notifications (sometimes known as ecological momentary interventions) are programmed messages sent to a user by the app and are commonly employed within behavior change apps to both monitor and provide support to people at risk of harmful alcohol consumption [3,10,59]. Push notifications are a time-varying component of *Drink Less* that can be further optimized to become just-in-time adaptive interventions that rely on decision rules in the provision of real-time support and can learn and adapt to the contextual and psychological circumstances of individuals over time [59]. Previous research has found that notifications are important components that influence engagement with behavior change apps [60-62]. This includes an ecological momentary assessment study with *Drink Less*, which found that establishing a daily routine is important for maintaining engagement and that the daily push notification supports such routines [63]. This study also found that time-varying, endogenous factors of motivation and perceived usefulness of the app were the most consistent predictors of engagement.

Our analysis suggested an approximate adjusted four-fold increase in the probability of using the app in the immediate hour following the notification (11 AM to noon) compared with the preceding hour (10 AM to 11 AM). For the 1 in 5 users belonging to the slow disengagers group, two interesting findings emerged. Firstly, the association between the notification and opening *Drink Less* in the subsequent hour decreased over time, and secondly, patterns of engagement for this group show, on average, a high probability of use over the first week but low probability after the second week. A possible reason for this decline in probability of use and association of the notification and use could be habituation to the daily notification, or turning the notification off. Importantly, we hypothesize that optimizing the notification policy may generate higher rates of engagement for this group.

### Future Research to Understand and Optimize the Notification Policy

To carefully create decision rules for the policy to evolve from an ecological momentary intervention to a just-in-time adaptive intervention, we will undertake a micro-randomized trial (MRT). The aim of the MRT is to further develop the push notification policy to improve engagement by targeting internal or external contextual circumstances that either influence excessive drinking (states of vulnerability) or events of engagement with the app (states of acceptability and opportunity) [64]. Visualization of engagement data helped inform the design of our MRT.

Table 5 summarizes how the visualizations from this exploratory research informed the design of our forthcoming MRT.

Primarily, this research guided our decision to shift the delivery time from 11 AM to 8 PM to exploit the potential increase in vulnerability to excess drinking, in an opportune and acceptable moment to engage with *Drink Less* [64]. To avoid the risk of an underpowered MRT, the expected effect size used in the sample size calculation of our MRT is based on a more conservative model, with the control defined as use between 9 AM and 11 AM and the treatment defined as use between 11 AM and 1 PM. This means that the MRT is powered to detect a marginal effect, quantified as a risk ratio, of sending a notification (compared with sending no notification) of 2.16 on user engagement rather than 4.22. We added 2 parallel arms to the MRT to provide an assessment of how engagement with *Drink Less* evolves over time when no notifications are provided and an exchangeable sample to compare the current policy of delivering a fixed notification daily, to a random notification policy, varying the content and sequence of notifications.

**Table 5:** Linking visualization to the design of a micro-randomized trial.

What we learnt from these analyses	Which visualization or analyses showed us this	How this informed the design of our randomized trial
The present notification appears to be a key driver of engagement	Figure 1, plot A: heat map of total sessions. Figure 1, plot B: heat map of total time on app (hours) Table 4: estimated risk ratio with 95% CI for the associations between exposure to the notification and app use within each cluster, at 3 time points (days 1, 7, and 30)	We chose to undertake a micro-randomized trial to both understand the causal effect of the notification on engagement, and to further optimize the delivery of notifications with respect to time-varying covariates, notifications, and outcomes
The impact of the notification seems to be strongest in the hour preceding delivery	Figure 1, plot A: heat map of total sessions	We set the time window to measure the proximal (ie, near-term) effect as 1 hour after delivery
Evenings seem to be an opportune and acceptable moment to engage with *Drink Less.* It is also a time of increased vulnerability to excess drinking	Figure 1, plot B: heat map of total time on app (hours)	We moved the delivery time of the notification to 8 PM
The notification may encourage the reporting of *alcohol-free days* more than *drink consumed*. This may be due to competing pressures for time at 11 AM	Figure 3: frequency distributions of when alcohol-free days and alcohol units are recorded	We intervened in the evenings to see if this is a more acceptable and opportune time to report drinks consumed
The notification may reduce the median time per session during the reminder of the day	Figure 2: line plot of median time spent on app (seconds)	We included a *no-notification* arm in our trial to capture a momentary assessment of engagement when no notifications are sent
The depth of engagement with *Drink Less* is low	Multimedia Appendix 2: summaries of use by module for all users	We trialed new notifications which target the perceived usefulness of *Drink Less* to encourage broader engagement
Slow disengagers (3679/19,233, 19.13%) have a high probability of engagement during the first week, but by day 30, this group has a low probability, suggesting a loss of motivation	Figure 4: probability of use on day after download by cluster group	We tested 30 new messages to increase novelty and motivation to remain engaged with *Drink Less* (Multimedia Appendix 8)
Exogenous impacts, such as public health campaigns, are likely to influence the cohort of users over time	Figure 6: time to disengagement (defined as the first day of 7 or more consecutive days of no use) by the engagement cluster	We included a *standard app version* arm in the trial, to provide an exchangeable sample to compare the fixed and random notification policies

### Limitations

This paper details exploratory research. Our estimates of the association between the notification and opening *Drink Less* do not represent a causal effect on engagement, as we are unable to account for systematic differences in use between the 2 periods that are unrelated to the notification. A randomized trial will allow for the causal effect of the notification to be understood. We also found that simple, accessible visualizations achieved our goal of understanding important patterns of engagement; however, when managing denser streams of data, more complex visualizations may be required.

An additional limitation is that disengagement is defined as a period of no use for 7 or more consecutive days and is considered as a one-time event instead of a repeated event; hence, the Kaplan-Meier plots are interpreted for the survival event *disengagement for the first time*. However, a proportion of users repeatedly disengage and then re-engage with *Drink Less*. It is not uncommon that even after disengaging a number of times, users re-engage for long, continuous spells of use with *Drink Less.* We aim to explore this in future research by visualizing the nature of repeated reengagement with accessible graphical applications and available shared toolsets [34]. An additional limitation is that we did not track whether users subsequently turned off or altered the delivery time of their notifications.

### Conclusions

Identifying patterns of engagement from voluminous, temporally dense data presents various challenges for researchers and practitioners. The summarization of such data with heat maps, timeline plots, and Kaplan-Meier plots can provide a clear picture of daily, weekly, and long-term patterns of use over time with a behavior change app. Optimizing engagement is a priority for many behavior change apps, and these visualizations provide a way to identify the key features of how this version of a behavior change app is engaged with.

For *Drink Less*, we have demonstrated the important role of visualizations by showing how these clearly identified how behavioral engagement varies over the day of the week and hour of the day, along with when users first disengage. The visualizations revealed that the daily notification is likely to strongly influence engagement with *Drink Less*. Both the average probability of use over 30 days and the association between use and the notification remained high for users in the engagers cluster yet steadily declined over time for users in the slow disengagers cluster. This suggests that a fixed notification policy can be effective for maintaining engagement for some users but ineffective for others. It is now our priority to understand the causal effect of the notification on engagement and to consider further optimizing the push notification policy to contextual circumstances of individuals over time to inform the development of a just-in-time adaptive intervention. The MRT aims to inform the development of decision rules to tailor the notification policy to individuals over time, with details found in our protocol [65].

## Appendix

Multimedia Appendix 1 Privacy notice.

Multimedia Appendix 2 Summaries of Use by Module for all users (n=19,233).

Multimedia Appendix 3 3D rotating heatmap of when sessions begin.

Multimedia Appendix 4 3D rotating graph total time on app per session.

Multimedia Appendix 5 Plot D animated over time.

Multimedia Appendix 6 Heat map of when downloads occur.

Multimedia Appendix 7 Plots of the elbow method and silhouette method.

Multimedia Appendix 8 Notification content of new message bank to be trialled in the micro-randomized trial.

## Abbreviations

AUDIT: Alcohol Use Disorders Identification Test
MRT: micro-randomized trial
NIHR: National Institute for Health Research
UKCTAS: UK Centre for Tobacco and Alcohol Studies
