# Supplementary material for: Engagement With a Behavior Change App for Alcohol Reduction: Data Visualization for Longitudinal Observational Study
Source: J Med Internet Res. 2020 Dec 11;22(12):e23369. doi: 10.2196/23369 (PMC7762688; doi:10.2196/23369)
Supplement: Multimedia Appendix 2 [file jmir_v22i12e23369_app2.docx]

#### Appendix Two: Summaries of Use by Module for all users (n=19,233).

| **Module** | **Percentage of screen views % (N)** | **Percentage time % (minutes)** |
| --- | --- | --- |
| Self-Monitoring and Feedback | 85 (7,095,348) | 80 (1,125,941) |
| Normative Feedback | 3 (245,426) | 2 (28,889) |
| Registration | 2 (190,430) | 5 (67,119) |
| Goal Setting and Feedback | 2 (172,124) | 3 (44,377) |
| Game (Cognitive Bias Re-training) | 2 (162,064) | 4 (59,950) |
| Identity Change | 1 (99,282) | 2 (26,830) |
| Action Planning | 1 (42,421) | 1 (11,397) |
| Other | <0.5 (35,720) | 1 (11,207) |
| Missing | 3 (297,190) | 2 (28,376) |
| **Total** | **100 (8,340,005)** | **100 (1,404,086)** |
