# Supplementary figures and images for: Engagement With a Behavior Change App for Alcohol Reduction: Data Visualization for Longitudinal Observational Study
Source: J Med Internet Res. 2020 Dec 11;22(12):e23369. doi: 10.2196/23369 (PMC7762688; doi:10.2196/23369)

#### Appendix Six: Heat map of when downloads occur.


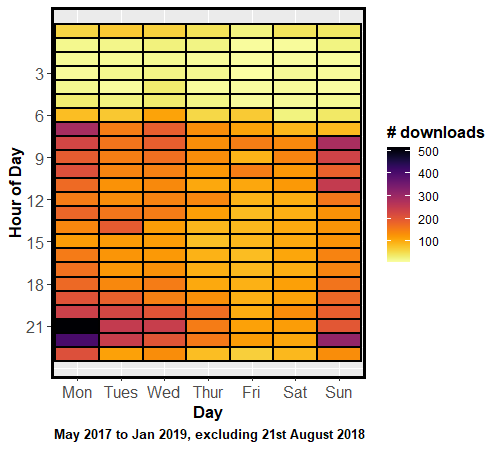

Supplement: Multimedia Appendix 6 [file jmir_v22i12e23369_app6.docx]

#### Appendix Seven: Plots of the Elbow Method and Silhouette Method.


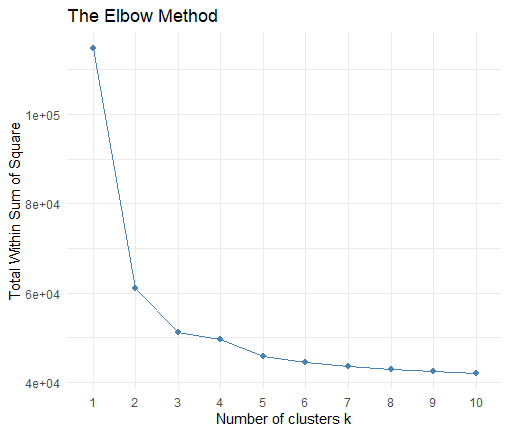


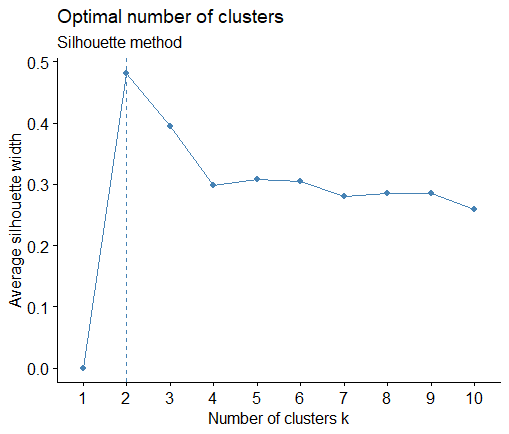

Supplement: Multimedia Appendix 7 [file jmir_v22i12e23369_app7.docx]
